# Supplementary figures and images for: Environmental and individual determinants of burrow-site microhabitat selection, occupancy, and fidelity in eastern chipmunks living in a pulsed-resource ecosystem
Source: PeerJ. 2023 Mar 23;11:e15110. doi: 10.7717/peerj.15110 (PMC10040179; doi:10.7717/peerj.15110)

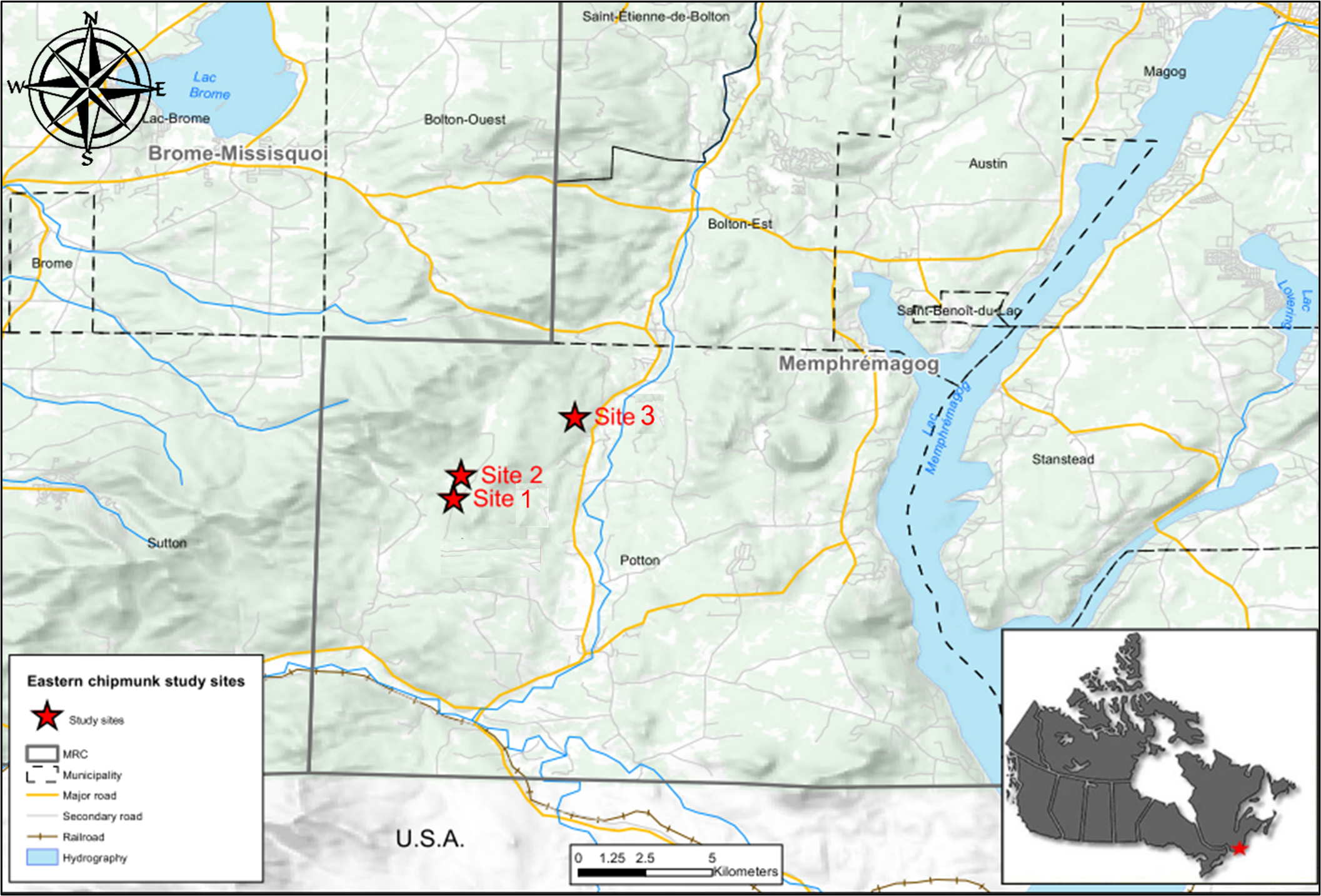

Supplement: Supplemental Information 1 [file peerj-11-15110-s001.png]

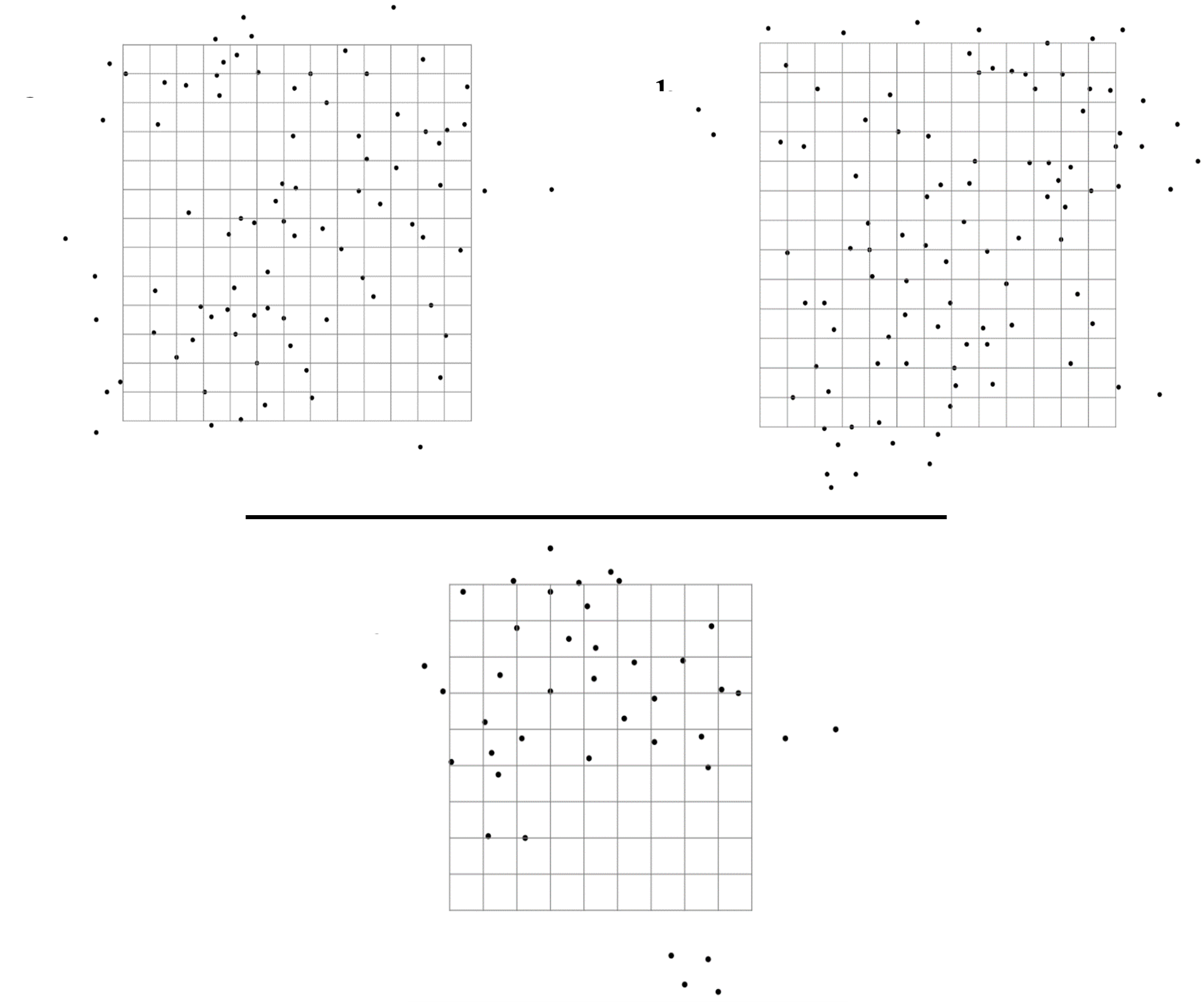

Supplement: Supplemental Information 2 — (A) Site 1, 260 m × 260 m. (B) Site 2, 260 m × 260 m. (C) Site 3, 180 m × 180 m. Each grid cell is 20 m × 20 m. [file peerj-11-15110-s002.png]

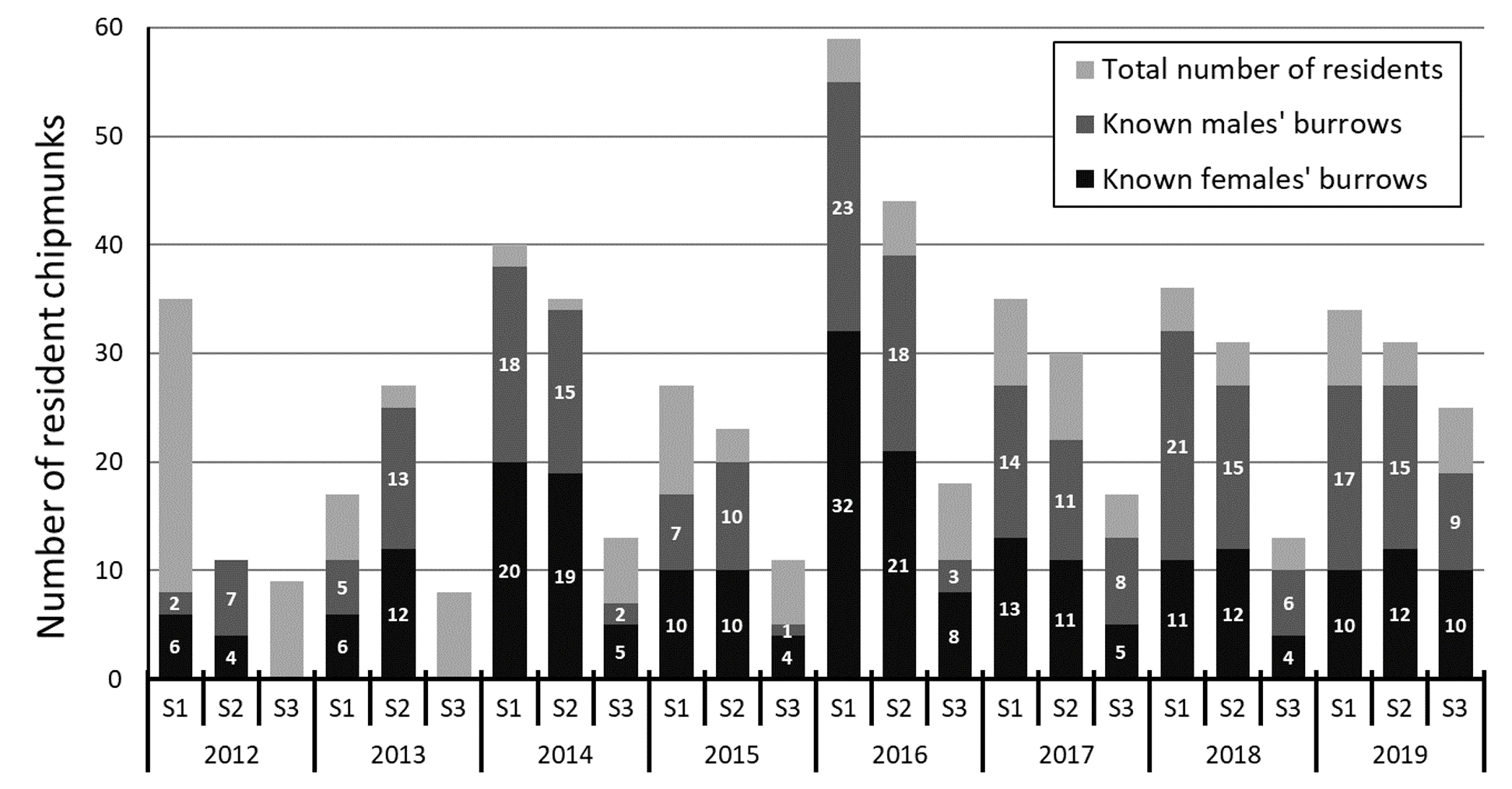

Supplement: Supplemental Information 3 — Individuals were considered residents when captured more than 5 times during a season and over a period longer than two weeks. S1 = Site 1, S2 = Site 2, S3 = Site 3. [file peerj-11-15110-s003.png]

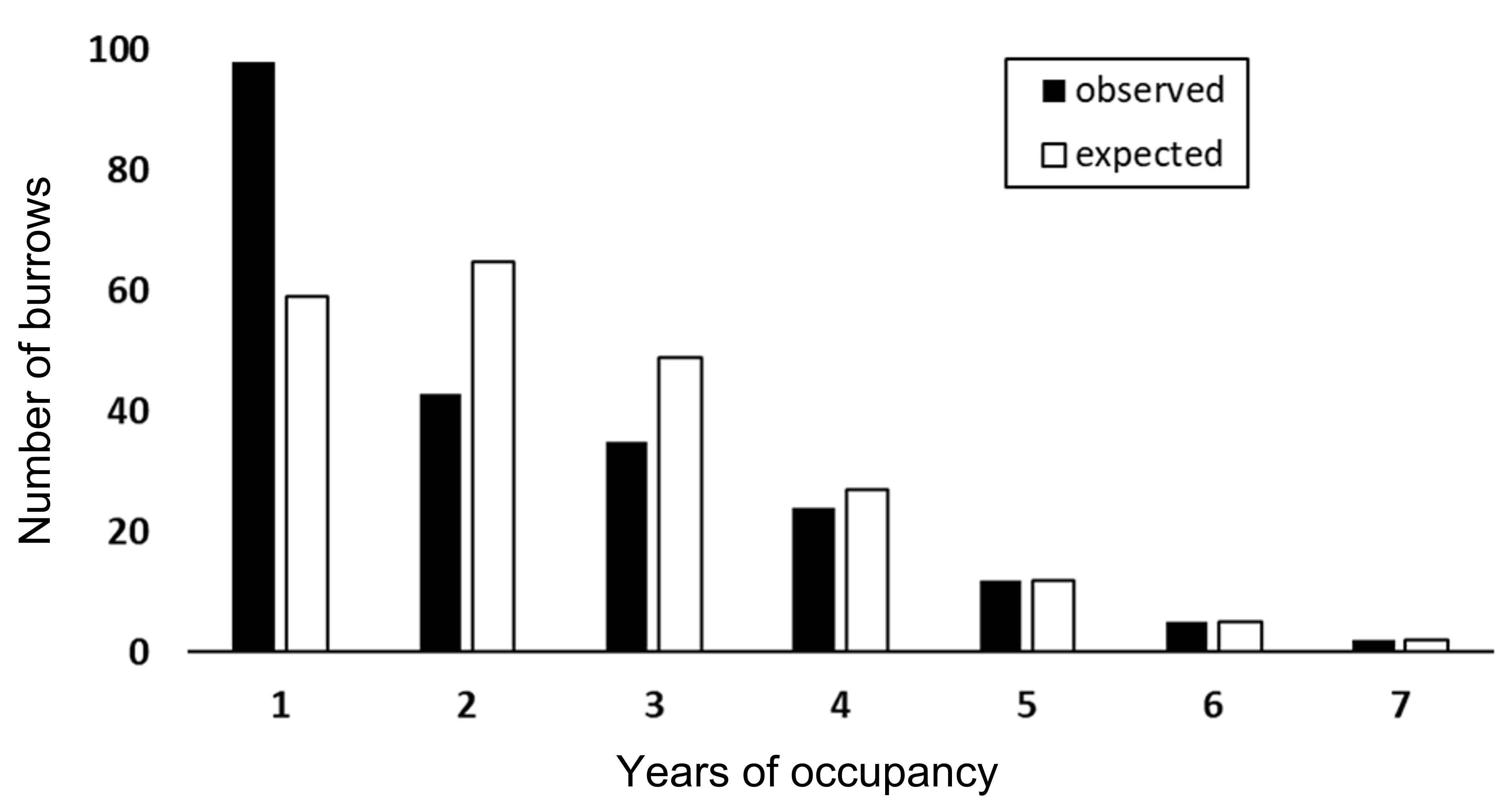

Supplement: Supplemental Information 4 — The expected frequencies were estimated from a zero-truncated Poisson distribution. Comparison between observed and expected frequencies was performed using a Fisher’s exact test: $\chi^2$χ25 = 37.56, P = 0.007, two-sided). [file peerj-11-15110-s004.png]

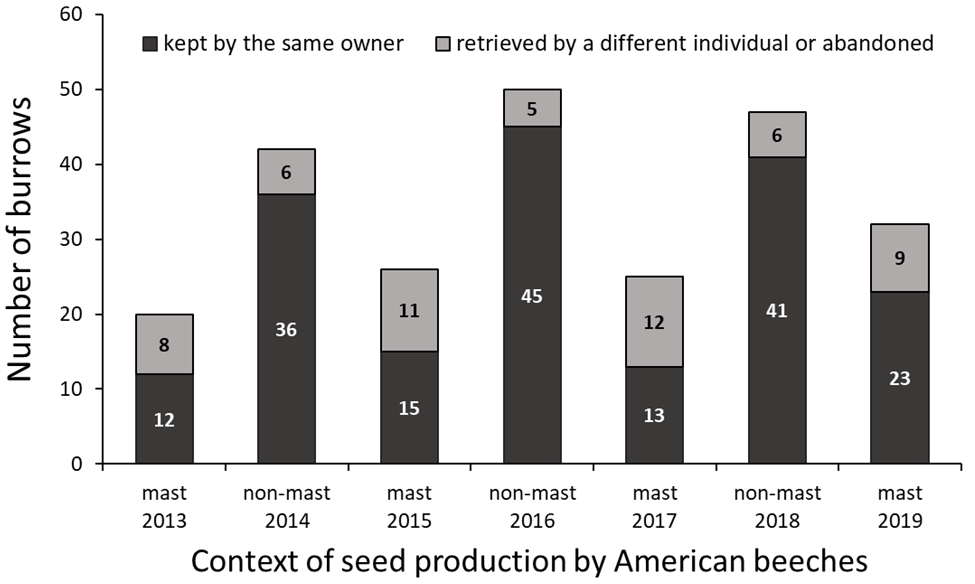

Supplement: Supplemental Information 5 [file peerj-11-15110-s005.png]

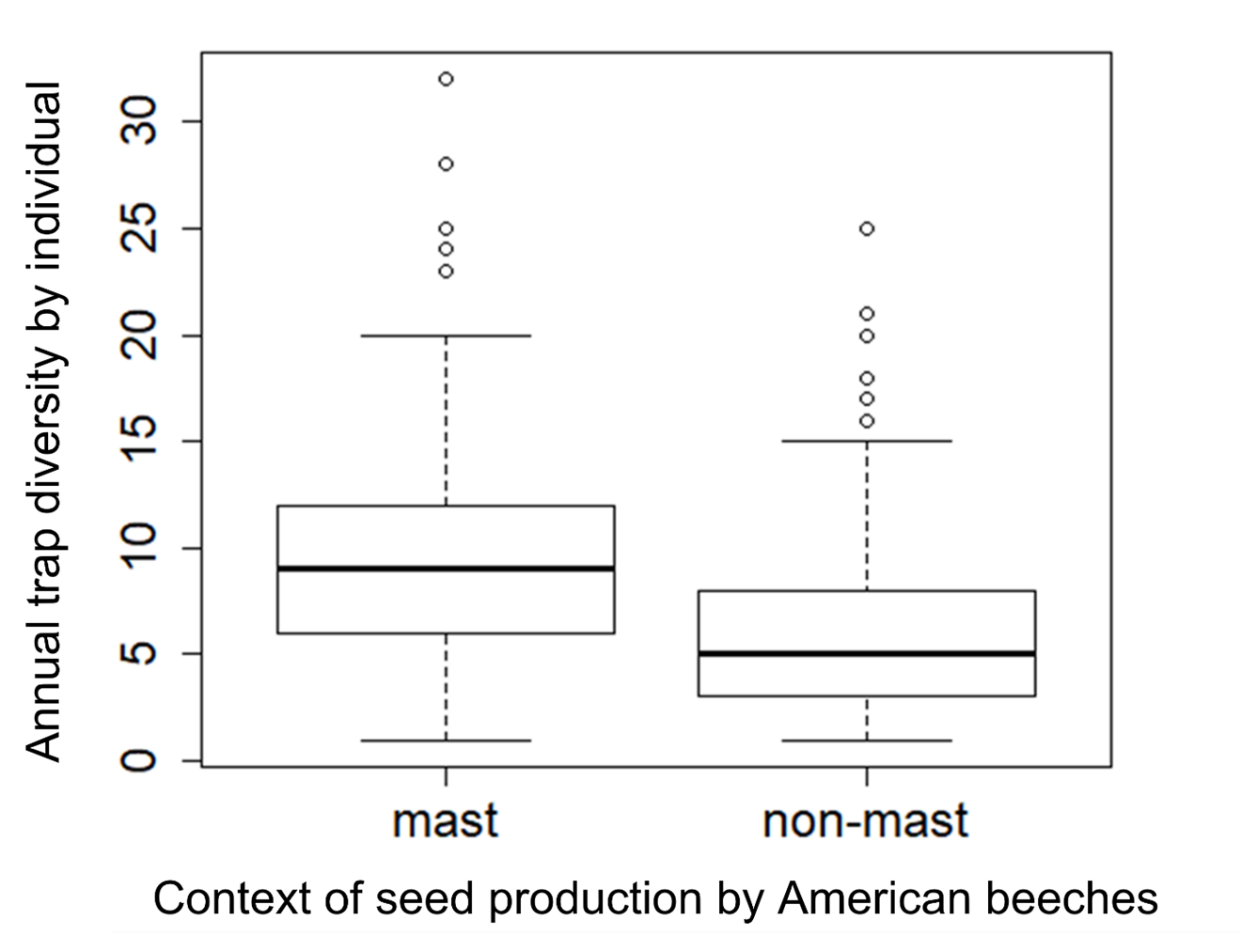

Supplement: Supplemental Information 6 — N mast = 4 years, 219 individuals, N non-mast = 4 years, 274 individuals. Boxes represent the first (lower limit) and third (upper limit) quartiles. The distributions’ median is illustrated by a black line. We performed a Student’s t test; t = 8.83, P < 0.001. [file peerj-11-15110-s006.png]
