# Supplementary material for: Environmental and individual determinants of burrow-site microhabitat selection, occupancy, and fidelity in eastern chipmunks living in a pulsed-resource ecosystem
Source: PeerJ. 2023 Mar 23;11:e15110. doi: 10.7717/peerj.15110 (PMC10040179; doi:10.7717/peerj.15110)
Supplement: Supplemental Information 7 — Analysis included 190 burrows that were occupied at least once since 2012. Microhabitat environmental variables were measured within a 10 m-radius plot around burrows. Significant variables (α = 0.05) are in bold. [file peerj-11-15110-s007.docx]

| Effect | Estimate | SE | *\|z*\| | *P* |
| --- | --- | --- | --- | --- |
| Intercept | -0.804 | 1.045 | 0.77 | 0.44 |
| **Juvenile/adult occupants ratio** | **1.352** | **0.207** | **6.53** | **<0.001** |
| **Female/male occupants ratio** | **-0.395** | **0.176** | **2.25** | **0.025** |
| Canopy cover (%) | <0.001 | 0.007 | 0.01 | 0.99 |
| **Herbaceous plants and shrubs (<1m) cover (%)** | **-0.011** | **0.005** | **2.24** | **0.025** |
| Rocks cover (%) | 0.002 | 0.010 | 0.23 | 0.82 |
| Number of logs (>2m) | 0.009 | 0.024 | 0.36 | 0.72 |
| Number of refuges | 0.007 | 0.020 | 0.36 | 0.72 |
| **Horizontal openness** | **-0.226** | **0.082** | **2.77** | **0.006** |
| Canopy height | 0.007 | 0.158 | 0.05 | 0.96 |
| Small hardwood tree density | 0.003 | 0.002 | 1.87 | 0.061 |
| Average DBH of large seed producing trees | 0.015 | 0.024 | 0.61 | 0.54 |
| Large beech trees density | -0.021 | 0.023 | 0.92 | 0.36 |
| Large sugar maple trees density | -0.018 | 0.021 | 0.85 | 0.40 |
| Large red maple trees density | -0.012 | 0.029 | 0.42 | 0.68 |
| Yellow trout lily cover (%) | 0.008 | 0.007 | 0.94 | 0.35 |
| Carolina spring beauty cover (%) | 0.016 | 0.024 | 0.66 | 0.51 |

*Notes:* The table presents all variables included in the full model, with their beta-coefficient, SE, *z* and *P* values. Small trees have a diameter at breast height (DBH) ≤ 10 cm, while large trees have a DBH > 31 cm. Horizontal openness was scored on a scale from 0 to 4, 0 being very open (very thin or no understory, easy to walk through) and 4 very close (dense understory, difficult to walk through). Seed-producing trees include American beech (*Fagus grandifolia*), red maple (*Acer rubrum*) and sugar maple (*A. saccharum*).
