# Supplementary material for: Environmental and individual determinants of burrow-site microhabitat selection, occupancy, and fidelity in eastern chipmunks living in a pulsed-resource ecosystem
Source: PeerJ. 2023 Mar 23;11:e15110. doi: 10.7717/peerj.15110 (PMC10040179; doi:10.7717/peerj.15110)
Supplement: Supplemental Information 8 — Analysis included 127 burrows that were occupied by adults only during the study period. Microhabitat environmental variables were measured within a 10 m-radius plot around burrows. Significant variables (α = 0.05) are in bold. [file peerj-11-15110-s008.docx]

| Effect | Estimate | SE | *\|z*\| | *P* |
| --- | --- | --- | --- | --- |
| Intercept | 0.489 | 1.982 | 0.25 | 0.81 |
| Canopy cover (%) | -0.012 | 0.014 | 0.90 | 0.37 |
| Herbaceous plants and shrubs (<1m) cover (%) | -0.008 | 0.008 | 0.92 | 0.36 |
| Rocks cover (%) | -0.016 | 0.019 | 0.87 | 0.38 |
| Number of logs (>2m) | 0.050 | 0.050 | 0.99 | 0.32 |
| Number of refuges | -0.074 | 0.040 | 1.83 | 0.068 |
| Horizontal openness | -0.250 | 0.162 | 1.55 | 0.12 |
| **Canopy height** | **0.824** | **0.310** | **2.66** | **0.008** |
| Small hardwood tree density | 0.004 | 0.003 | 1.24 | 0.22 |
| Average DBH of large seed-producing trees | 0.055 | 0.052 | 1.05 | 0.29 |
| Large beech trees density | -0.037 | 0.042 | 0.90 | 0.37 |
| Large sugar maple trees density | -0.022 | 0.039 | 0.57 | 0.57 |
| Large red maple trees density | -0.095 | 0.057 | 1.69 | 0.092 |
| **Yellow trout lily cover (%)** | **-0.035** | **0.014** | **2.47** | **0.014** |
| Carolina spring beauty cover (%) | -0.030 | 0.080 | 0.38 | 0.71 |

*Notes:* The table presents all variables included in the full model, with their beta-coefficient, SE, *z* and *P* values. Horizontal openness was scored on a scale from 0 to 4, 0 being very open (very thin or no understory, easy to walk through) and 4 very close (dense understory, difficult to walk through). Small trees have a diameter at breast height (DBH) ≤ 10 cm, while large trees have a DBH > 31 cm. Seed-producing trees include American beech (*Fagus grandifolia*), red maple (*Acer rubrum*) and sugar maple (*A. saccharum*).
