# Supplementary material for: Environmental and individual determinants of burrow-site microhabitat selection, occupancy, and fidelity in eastern chipmunks living in a pulsed-resource ecosystem
Source: PeerJ. 2023 Mar 23;11:e15110. doi: 10.7717/peerj.15110 (PMC10040179; doi:10.7717/peerj.15110)
Supplement: Supplemental Information 9 — Analysis included 217 instances of consecutive occupancy events from 2012 to 2019. Significant variables (α = 0.05) are in bold. [file peerj-11-15110-s009.docx]

| Effect | Estimate | SE | *\|z*\| | *P* |
| --- | --- | --- | --- | --- |
| Intercept | -10.190 | 4.158 | 2.45 | 0.014 |
| Sex | -0.347 | 0.425 | 0.82 | 0.41 |
| **Age class** | **0.491** | **0.249** | **1.97** | **0.049** |
| Cohort - summer | 0.199 | 0.442 | 0.45 | 0.65 |
| **Mast** | **1.805** | **0.491** | **3.68** | **<0.001** |
| Weight (averaged over the active season) | 0.059 | 0.032 | 1.82 | 0.068 |
| Number of neighbors | 0.016 | 0.066 | 0.24 | 0.81 |
| **Canopy cover** | **0.068** | **0.026** | **2.66** | **0.008** |
| Herbaceous plants and shrubs (<1m) cover (%) | 0.012 | 0.013 | 0.87 | 0.38 |
| Rocks cover (%) | -0.027 | 0.030 | 0.89 | 0.38 |
| Number of logs (>2m) | 0.089 | 0.079 | 1.13 | 0.26 |
| Number of refuges | -0.027 | 0.051 | 0.53 | 0.60 |
| Horizontal openness | 0.024 | 0.233 | 0.10 | 0.92 |
| Canopy height | -0.190 | 0.464 | 0.41 | 0.68 |
| Small hardwood trees density | -0.001 | 0.004 | 0.17 | 0.87 |
| Average DBH of large seed-producing trees | -0.059 | 0.065 | 0.89 | 0.37 |
| Large beech trees density | -0.025 | 0.060 | 0.41 | 0.68 |
| Large sugar maple trees density | 0.011 | 0.063 | 0.18 | 0.86 |
| **Large red maple trees density** | **0.811** | **0.352** | **2.30** | **0.021** |

*Notes:* The table presents all variables included in the full model, with their beta-coefficient, SE, *z* and *P* value. Sex has two levels: female (reference) or male. Age class is an individual’s minimal age, in years, with 0 being the juvenile state. Cohort has two levels: spring (reference) and summer. Horizontal openness was scored on a scale from 0 to 4, 0 being very open (very thin or no understory, easy to walk through) and 4 very close (dense understory, difficult to walk through). Number of neighbors is the number of adjacent occupied home ranges overlapping a focal occupant’s home range on a given year, considering a mean home range size of 40 m in radius around a burrow on our study sites. Small trees have a diameter at breast height (DBH) ≤ 10 cm, while large trees have a DBH > 31 cm. Seed-producing trees include American beech (*Fagus grandifolia*), sugar maple (*Acer saccharum*) and red maple (*A. rubrum*).
